# Event and Comment

**Published:** 1908-03-15

**Authors:** 


### DENTAL REGISTER.

Vol. LXII. March 15, 1908. No. 3.

### EVENT AND COMMENT.

### Value of the Cast Inlay.

The constant search of the profession is to find a method which shall supplant the tedious and painstaking methods now employed in filling teeth, and this idea occupies so prominent a place in the minds of every operator of much experience that we were all prepared to accept anything which seemed likely to be a succedaneum. When the porcelain inlay was announced we were sure that the solution of our problem was in sight. But, alas, we have found that it takes infinite pains and great skill to satisfactorily manipulate this material, and that this tedious process is so expensive that many can not afford the cost, and besides there are many other complications that conspire to dampen the enthusiasm of the mass of practitioners. We believe the porcelain inlay filling has surely come to stay, but, like the continuous gum plate, it will never supplant present materials because few men will have the courage and high order of skill to comply with its exacting demands. Apparently the profession has dropped the porcelain inlay with a suddeness that is hardly justifiable and is making a mad scramble for the gold inlay, under the impression that this is a method that will work itself. All you have to do is to press the lever or touch the electric button and the Genii will do the rest. But already we are finding that every step in the casting of gold inlays must be taken with great precision to insure a successful result. The cavity preparation must be of ideal form, and the cavity margins and retention must be carefully planned and worked out. The wax model can not be carelessly made: it must reproduce the form and detail of the cavity precisely, and restore the tooth's contour and be capable of removal from the cavity without distorting it appreciably. Its investment in a proper material for casting is not to be carelessly done or it will be found that the casting will not fit the cavity. In casting the investment mould must be properly heated preparatory to the casting, and the proper degree of force must be applied to the melted gold and at just the right moment to displace the air in the mould, and to replace it with the melted metal. We know now that by overheating and cracking the mould, or by excessive force, the fused metal will be forced into the investment causing its distortion and thus producing such a casting as will not fit the cavity at all. Then comes the polishing and setting, which require also a good degree of skill and no little practice and patience. In fact we are assured from a somewhat limited use of the several methods that none of them will appeal to the careless workman, or to the man who is always in a hurry, so strongly as it is now thought. It has come to stay and is to be one of our reliable filling methods, but it will not soon supplant the older tried and proved filling materials. There can be no question but it will be greatly useful in crown and bridge work requiring skill.

### Cast Inlay Machines.

We have as yet had insufficient experience with the method of filling teeth with cast filings to enable anyone to speak confidently as to the possible value of this form of repairing the ravages of decay of the teeth. Immediately on the announcement of the new process, some of our Editors and not a few enthusiastic practitioners at once announced the milenium in dentistry. And the suspense put upon the profession by the inventor because he was not ready to supply a suitable casting machine was so intense, that every man who had an inventive turn of mind seems to have put it to work inventing machines, until the number and variety now on the market are so numerous that no one seems able to pick a winner. We have more than a dozen different machines using air pressure direct from the compressor, and twice as many more generating air or steam pressure by combustion, explosions, etc., to say nothing of the various forms of centrifugal machines from the spinning top to the complicated ball-bearing machine that costs fifty dollars. We certainly have worked our wits to good purpose, and there need be no need for any one to hesitate about taking up the work, because of lack of an appliance, as they can be had for the asking almost, and on up to a hundred dollars. We are often asked which one is the best to buy, and are compelled to decline the honor of even offering an opinion, as all seem to be able to produce results. And we have found from experience that one can not select instruments for another with much more success than he can horses or wives. We hear of one man who places the mould in a tin bucket which he swings around his head holding it by its bail. Another uses the coil spring in the end of a curtain roller effectively. And some use a piece of wet asbestos paper on the ball of the thumb to force the melted gold into the mould by the steam generated when the wet paper comes into contact with the hot gold. Of course all of these crude appliances are inadvisable because they are unreliable. We are learning that good castings can not be made carelessly and necessarily the more accurately adjusted and devised the apparatus the more uniform will be the results, and we shall not be surprised to find eventually, that more complicated and skillfully planed machines than we now have will be demanded before this work takes rank as a standard filling process.

